# The *ITGAV *rs3738919 variant and susceptibility to rheumatoid arthritis in four Caucasian sample sets

**DOI:** 10.1186/ar2828

**Published:** 2009-10-09

**Authors:** Jade E Hollis-Moffatt, Kerry A Rowley, Amanda J Phipps-Green, Marilyn E Merriman, Nicola Dalbeth, Peter Gow, Andrew A Harrison, John Highton, Peter BB Jones, Lisa K Stamp, Pille Harrison, B Paul Wordsworth, Tony R Merriman

**Affiliations:** 1 Department of Biochemistry, University of Otago, 710 Cumberland Street, Dunedin 9054, New Zealand; 2 Department of Medicine, University of Auckland, 2 Park Road, Auckland 1023, New Zealand; 3 Rheumatology, Middlemore Hospital, 100 Hospital Road, Auckland 2025, New Zealand; 4 Department of Medicine, University of Otago, 23A Mein Street, Wellington 6242, New Zealand; 5 Department of Medicine, University of Otago, 201 Great King Street, Dunedin 9016, New Zealand; 6 Department of Medicine, University of Otago, 2 Riccarton Avenue, Christchurch 8140, New Zealand; 7 Nuffield Department of Orthopaedics, Nuffield Orthopaedic Centre, Windmill Road, Oxford OX3 7LD, UK

## Abstract

**Introduction:**

Angiogenesis is an important process in the development of destructive synovial pannus in rheumatoid arthritis (RA). The *ITGAV *+gene encodes a cell cycle-associated antigen, integrin ανβ 3, which plays a role in RA angiogenesis. Previously, two independent studies identified an association between the major allele of the *ITGAV *single-nucleotide polymorphism (SNP) *rs3738919 *and RA. We therefore tested this association in an independent study using New Zealand (NZ) and Oxford (UK) RA case control samples.

**Methods:**

We compared genotype frequencies in 740 NZ Caucasian RA patients and 553 controls genotyped for *rs3738919*, using a polymerase chain reaction-restriction fragment length polymorphism assay. A TaqMan genotyping SNP assay was used to type 713 Caucasian RA patients and 515 control samples from Oxford for the *rs3738919 *variant. Association of *rs3738919 *with RA was tested in these two sample sets using the chi-square goodness-of-fit test. The Mantel-Haenszel test was used to perform a meta-analysis, combining the genetic results from four independent Caucasian case control cohorts, consisting of 3,527 cases and 4,126 controls. Haplotype analysis was also performed using SNPs *rs3911238*, *rs10174098 *and *rs3738919 *in the Wellcome Trust Case Control Consortium, NZ and Oxford case control samples.

**Results:**

We found no evidence for association between *ITGAV *and RA in either the NZ or Oxford sample set (odds ratio [OR] = 0.88, *P*_*allelic *_= 0.11 and OR = 1.18, *P*_*allelic *_= 0.07, respectively). Inclusion of these data in a meta-analysis (random effects) of four independent cohorts (3,527 cases and 4,126 controls) weakens support for the hypothesis that *rs3738919 *plays a role in the development of RA (OR_*combined *_= 0.92, 95% confidence interval 0.80 to 1.07; *P *= 0.29). No consistent haplotype associations were evident.

**Conclusions:**

Association of *ITGAV *SNP *rs7378919 *with RA was not replicated in NZ or Oxford case control sample sets. Meta-analysis of these and previously published data lends limited support for a role for the *ITGAV *in RA in Caucasians of European ancestry.

## Introduction

Rheumatoid arthritis (RA) is a common systemic autoimmune disease characterised by chronic synovial inflammation leading to the formation of invasive, destructive pannus. The extensive formation of new blood vessels within the affected joint (hyperangiogenesis) is an important component of pannus formation [1].

A heritable component to RA is supported by twin studies [2] and the markedly increased sibling recurrence risk (λ_s _= 5 to 7.2) compared with the general population [3]. Association with the *HLA-DRB1 *locus is well established, with many other loci also incriminated. These include the protein tyrosine phosphatase non-receptor 22 (*PTPN22*) gene [4,5], cytotoxic T-lymphocyte antigen 4 (*CTLA4*) [6], an intergenic region on human chromosome 6 [7,8], signal transducer and activator of transcription 4 (*STAT4*) [9], the tumour necrosis factor receptor-associated factor 1 region (*TRAF/C5*) [7,10,11] and CD40, CCL21 and IL2RB [12,13].

The integrin ανβ3 (*ITGAV*) locus contains 30 exons spanning more than 90 kb of genomic DNA on human chromosome 2q31. It encodes the αν subunit of the cell cycle-associated antigen, integrin ανβ3, which plays a major role in RA angiogenesis. Angiogenesis is stimulated in RA by the increased metabolic demand of the pathologically active synovial tissues [1,14-16]. Potentially, inhibition of this angiogenesis might suppress the destructive activities of pannus and even control disease activity [17]. This is supported by studies in animal models in which injection of ανβ3 antagonists has shown inhibition of neovascularisation and attenuation of joint inflammation [18].

A previous genome-wide linkage scan suggested 19 non-HLA regions contributing to RA in a French population [19]. One of these regions, on human chr2q31, contains the *ITGAV *gene (*CD51*). Subsequently, Jacq and colleagues [20] demonstrated an association between RA and the *ITGAV rs3738919 *C allele in a French Caucasian population (odds ratio for allele frequency difference [OR_*allelic*_] = 0.77, 95% confidence interval [CI] 0.63 to 0.94). Significant association with the *rs3738919 *C allele was also demonstrated from imputed data by the Wellcome Trust Case Control Consortium (WTCCC) (OR_*allelic *_= 0.91, 95% CI 0.83 to 1.00) [21]. Collectively, these studies suggest a role for *ITGAV *in RA and justify further investigation of this locus. We therefore tested this association in an independent study using New Zealand (NZ) and Oxford (UK) RA case control samples.

## Materials and methods

### Study subjects

The NZ population-based Caucasian sample consists of 740 RA patients fulfilling the American College of Rheumatology (ACR) criteria for RA [22]. Of the patients for whom data were available, 34.3% (234/683) were male, 82.9% (538/649) were rheumatoid factor (RF)-positive, 68.1% (275/404) were anti-cyclic citrullinated peptide (anti-CCP)-positive and 79.4% (576/725) carried the *HLA-DRB1 *shared epitope. Ethical approval for recruitment of cases was given by the New Zealand Multi-Region Ethics Committee, and recruitment of the controls was approved by the Lower South Ethics Committee. All patients provided written informed consent for the collection of samples and subsequent analysis. The control sample consisted of 553 NZ European Caucasians (226/552 male; 40.9%) with no history of autoimmune disease.

The 713 UK patients were recruited in Oxford, with informed consent from attendees at the rheumatology outpatient clinic at the Nuffield Orthopaedic Centre. All fulfilled the 1987 ACR criteria for RA; the average age of onset was 48 years, 28% were male, 77% were positive for RF and 77% carried the *HLA-DRB1 *shared epitope. Healthy ethnically matched controls (n = 515) were recruited from the same locale as that of blood donors. Approval for the study was given by the Oxford Research Ethics Committee (OxRec number C02.032).

### DNA extraction and genotyping

DNA was extracted from peripheral blood samples of the RA patients and controls by means of guanidine isothiocyanate-chloroform extraction methods. NZ study participants were genotyped for the *ITGAV *single-nucleotide polymorphism (SNP) *rs3738919 *using a polymerase chain reaction-restriction fragment length polymorphic SNP genotyping assay as follows: forward primer CACTTTCTGTAAATTAGTGTTAGATCAAAAGG and reverse primer GCTTATAACTCACAATTCAGATTTTTGCC (primers from Sigma-Genosys, Sydney, Australia). The C allele (major allele) of the *rs3738919 *product (286 base pairs) was digested using the *Alu*I restriction enzyme to form fragments of 223 and 63 base pairs. Oxford study participants were genotyped for *rs3738919 *using the TaqMan genotyping assay C___1278131_1, and both the NZ and Oxford samples were genotyped for the *rs10174098 *and *rs3911238 ITGAV *variants using the TaqMan genotyping assays C__30567648_10 and C___7617051_10, respectively, from Applied Biosystems (Scoresby, Australia).

Although the imputed *rs3738919 *data were available and were previously reported by Ahnert and Kirsten [21], we reimputed *rs3738919 *genotypes in order to provide information on imputation parameters. RA case and control genotypes were imputed from the WTCCC dataset using IMPUTE software [23]. Genotypes were imputed from 89% of cases (n = 1,659) and 90% of controls (n = 2,639) using a 7-Mb region and a calling threshold of 0.7.

### Statistical analysis

An *a priori *power calculation was made based on the combined French [20] and WTCCC [21] OR (OR_*allelic *_= 1.16), using 800 cases and 600 controls with a major allele frequency of 65%. The power to detect association of *rs3738919 *to RA using either the NZ or Oxford sample set was estimated to be 47%, using α = 0.05. *ITGAV *SNPs were tested for deviation from Hardy-Weinberg equilibrium in both the control and RA samples using a chi-square goodness-of-fit test. The significance of differences in the minor allele frequency between RA patients and controls, and stratified patients, was assessed using the chi-square goodness-of-fit test.

Of the five *ITGAV *SNPs (*rs3911238*, *rs2887827*, *rs10174098*, *rs13006571 *and *rs16828163*) genotyped by the WTCCC and *rs3738919*, only three (*rs3911238*, *rs10174098 *and *rs3738919*) were required to tag all major haplotypes defined by the six variants. Haplotype association analysis was performed using the SHEsis software package [24], which uses a full-precise-iteration algorithm to construct haplotypes and estimate haplotype frequencies.

Meta-analysis combining the French [20], WTCCC [25], NZ and Oxford samples was performed using STATA version 8.0 (StataCorp LP, College Station, TX, USA). The Mantel-Haenszel test was used to estimate the average conditional common OR between the four independent sample sets and to test for any heterogeneity between the four groups using both fixed and random effects. R software [26] was used to perform the Cochran-Armitage trend test to determine recessive, additive and dominant trend values (Table 1).

**Table 1:** Allele and genotype distribution of *rs3738919*

Cohort	Case, number (frequency)	Control, number (frequency)	*P *value	OR (95% CI)
New Zealand				
Minor allele	501 (0.339)	408 (0.369)	0.11	0.88 (0.75-1.03)
Genotype 1,1	326 (0.38)	210 (0.38)	-	1
1,2	327 (0.44)	278 (0.50)	0.021	0.76 (0.60-0.96)
2,2	87 (0.12)	65 (0.12)	0.43	0.86 (0.60-1.24)
HWE	0.72	0.061		
Dominant			0.028	
Additive			0.10	
Recessive			0.99	
Oxford, UK				
Minor allele	506 (0.355)	329 (0.319)	0.068	1.17 (0.99-1.39)
Genotype 1,1	300 (0.42)	235 (0.46)	-	1
1,2	319 (0.45)	231 (0.45)	0.52	1.08 (0.85-1.38)
2,2	93 (0.13)	49 (0.10)	0.044	1.49 (1.01-2.19)
HWE	0.60	0.47		
Dominant			0.22	
Additive			0.069	
Recessive			0.055	
WTCCC				
Minor allele	1,141 (0.344)	1,928 (0.365)	0.044	0.91 (0.83-1.00)
Genotype 1,1	708 (0.43)	1,044 (0.40)	-	1
1,2	761 (0.46)	1,262 (0.48)	0.079	0.89 (0.78-1.01)
2,2	190 (0.11)	333 (0.13)	0.094	0.84 (0.69-1.03)
HWE	0.50	0.11		
Dominant			0.043	
Additive			0.041	
Recessive			0.26	
Jacq *et al*. [20]				
Minor allele	292 (0.352)	343 (0.413)	0.01	0.77 (0.63-0.94)
Genotype 1,1	166 (0.40)	148 (0.36)	-	1
1,2	206 (0.50)	191 (0.46)	0.80	0.96 (0.71-1.29)
2,2	43 (0.10)	76 (0.18)	0.002	0.50 (0.33-0.78)
HWE	0.0718	0.30		
Dominant			0.20	
Additive			0.0096	
Recessive			0.0011	
Combined				
Minor allele	2,440 (0.346)	3,008 (0.365)	0.014	0.92 (0.86-0.98)
Genotype 1,1	1,500 (0.43)	1,637 (0.40)	-	1
1,2	1,614 (0.46)	1,966 (0.48)	0.028	0.90 (0.81-0.99)
2,2	413 (0.12)	523 (0.13)	0.046	0.86 (0.74-1.00)
HWE	0.51	0.083		
Dominant			0.011	
Additive			0.013	
Recessive			0.20	

CI, confidence interval; HWE, Hardy-Weinberg equilibrium *P *value; OR, odds ratio; WTCCC, Wellcome Trust Case Control Consortium.

## Results

### Analysis of *rs3738919 *in New Zealand and Oxford Caucasian rheumatoid arthritis samples

We genotyped *rs3738919 *across the NZ and Oxford RA case control cohorts and found no evidence for an association between *ITGAV *and RA (*P*_*allelic *_= 0.11; OR_*allelic *_= 0.88 [95% CI 0.75 to 1.03] and *P*_*allelic *_= 0.07; OR_*allelic *_= 1.17 [95% CI 0.99 to 1.39], respectively) (Table 1). The direction of the NZ allele distribution is consistent with the previous French [20] and WTCCC [21] data, with the minor allele under-represented in the case groups in all three cohorts (Table 1). However, the allele distribution in the Oxford RA cohort differs in that the minor allele is over-represented in the patient group compared with the control group (Table 1).

### Haplotype analysis

Association analysis using our imputed genotypes for *rs3738919 *(*P *= 0.04) from the WTCCC data were consistent with the previous report of an association of this SNP with RA in the WTCCC [21]. In our analysis, we obtained imputed genotypes from 89% of the WTCCC case control subjects. Presumably, this reflects the influence of the low linkage disequilibrium that exists between *rs3738919 *and the genotyped SNPs (0.01 <*r*^2 ^< 0.54 in CEU [Centre d'Etude du Polymorphisme Humain Utah] HapMap [27]) on confidence calls of imputed genotypes. Haplotype analysis (Table 2) was performed using the actual genotypes from *ITGAV *SNPs *rs3911238 *and *rs10174098 *(Additional data file 1) and the imputed genotypes for *rs3738919 *(Table 1). One susceptibility haplotype (1-1-1), containing the major allele at each of the three SNPs, was significantly over-represented in cases compared with controls (OR = 1.15, 95% CI 1.02 to 1.30; *P *= 0.021).

**Table 2:** Three-marker haplotype analysis of *ITGAV *single-nucleotide polymorphisms (*rs10174098*, *rs3911238*, *rs3738919*) in the Wellcome Trust Case Control Consortium, New Zealand and Oxford sample sets

*rs10174098*	*rs3911238*	*rs3738919*	Case, number (frequency)	Control, number (frequency)	*P *value	OR (95% CI)
WTCCC						
1	1	2	866 (0.261)	1,451 (0.275)	0.19	0.94 (0.85-1.03)
2	1	1	858 (0.259)	1,382 (0.262)	0.82	0.99 (0.90-1.09)
1	2	1	792 (0.239)	1,226 (0.232)	0.43	1.04 (0.94-1.16)
1	1	1	524 (0.158)	741 (0.140)	0.021	1.15 (1.02-1.30)
2	1	2	219 (0.066)	405 (0.077)	0.070	0.85 (0.72-1.01)
New Zealand						
1	1	2	357 (0.244)	260 (0.263)	0.29	0.90 (0.75~1.09)
2	1	1	364 (0.249)	240 (0.243)	0.73	1.03 (0.86~1.25)
1	2	1	359 (0.246)	223 (0.225)	0.26	1.12 (0.92~1.35)
1	1	1	241 (0.165)	170 (0.172)	0.60	0.95 (0.76~1.17)
2	1	2	110 (0.075)	73 (0.074)	0.91	1.02 (0.75~1.39)
Oxford, UK						
1	1	2	310 (0.256)	252 (0.253)	0.80	1.03 (0.85-1.24)
2	1	1	306 (0.253)	262 (0.262)	0.67	0.96 (0.79-1.16)
1	2	1	335 (0.277)	251 (0.252)	0.16	1.15 (0.95-1.39)
1	1	1	158 (0.130)	162 (0.162)	0.04	0.78 (0.62-0.99)
2	1	2	78 (0.064)	57 (0.057)	0.47	1.14 (0.80-1.62)

Only haplotypes with frequency of at least 0.05 in controls are shown. CI, confidence interval; HWE, Hardy-Weinberg equilibrium; OR, odds ratio; WTCCC, Wellcome Trust Case Control Consortium.

Variants *rs3911238 *and *rs10174098 *were typed over the NZ and Oxford sample sets, and association of three-marker haplotypes was examined (Additional data file 1 and Table 2). There was no support for a positive association of the 1-1-1 haplotype with RA in either the NZ or Oxford sample set. There was a significant protective effect for this haplotype in the Oxford samples (OR = 0.78, 95% CI 0.62 to 0.99; *P *= 0.042).

### Meta-analysis of *ITGAV *in the four independent case control sample sets

**Figure 1:**
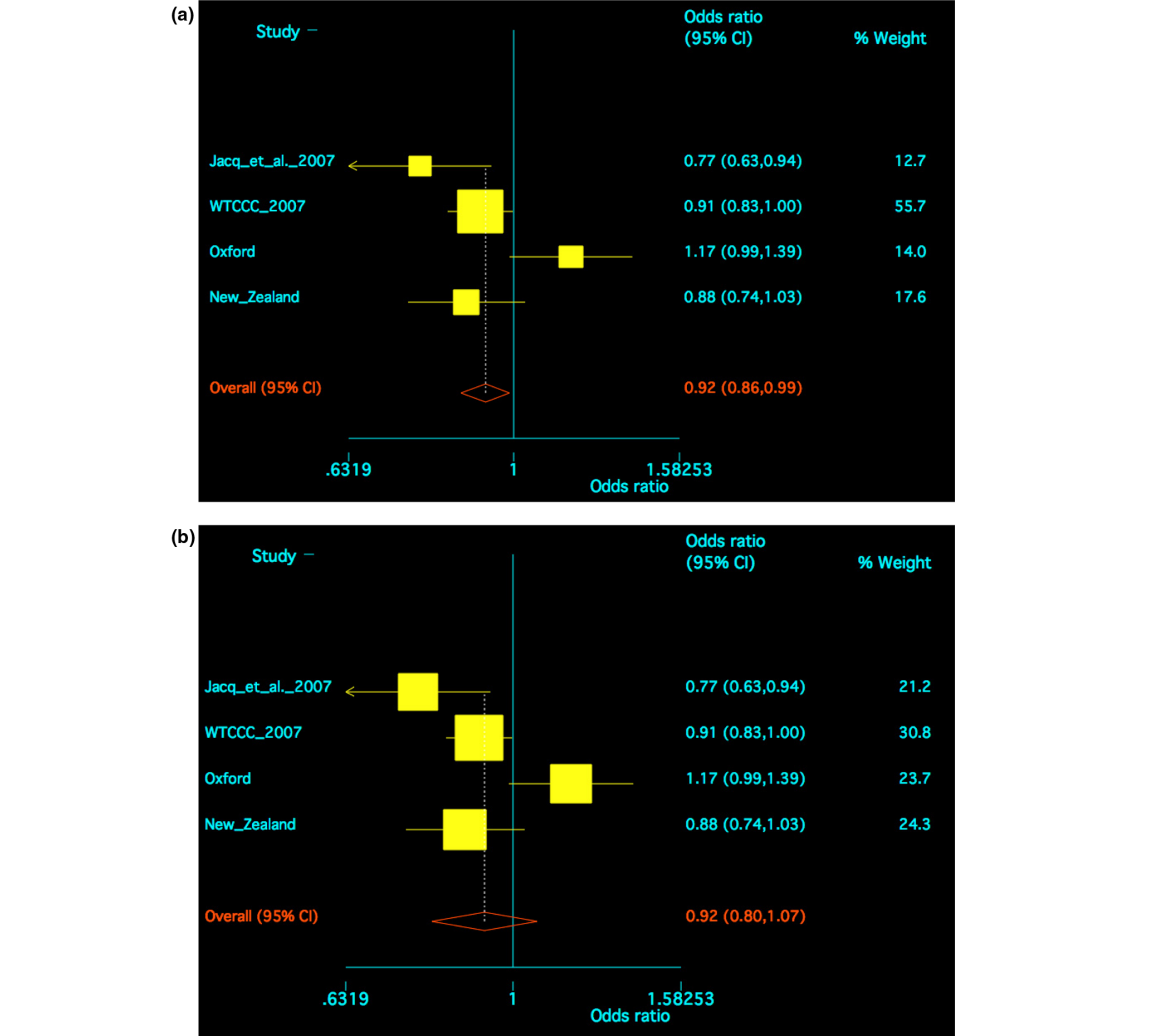
Meta-analysis of the *ITGAV *single-nucleotide polymorphism *rs3738919 *in four independent rheumatoid arthritis Caucasian cohorts: Jacq and colleagues [20], Wellcome Trust Case Control Consortium (WTCCC) [25], New Zealand and Oxford sample sets. **(a) **Fixed effects model. **(b) **Random effects model. CI, confidence interval.

Meta-analysis of all four sample sets, using a fixed effects model, revealed some evidence for an association of *rs3738919 *with RA (OR = 0.92, 95% CI 0.86 to 0.99; *P *= 0.021) (Figure 1a). Because of evidence for heterogeneity between the sample sets (Breslow-Day *P *= 0.011), a random effects model was also used for meta-analysis; this did not provide evidence for an association of *rs3738919 *with RA (OR = 0.92, 95% CI 0.80 to 1.07; *P *= 0.29) (Figure 1b). The Oxford sample set is significantly different from the other three sample sets at *rs3738919*; however, the Oxford patient sample set did not have any large differences in relation to gender (Oxford 28% male, NZ 34%, WTCCC 25%, France 13%), RF status (Oxford 77% positive, NZ 83%, WTCCC 84%, France 75%) or inheritance of the shared epitope (Oxford 77% positive, NZ 79%, WTCCC 79%, France 79%).

### Stratification according to subphenotype

The NZ and Oxford samples were stratified according to gender, RF and shared epitope status (Table 3). The stratification results did not reveal any significant differences in the *rs3738919 *genotype distribution for RA patients. There was a difference in the *rs3738919 *genotype distribution of male and female patients in the NZ cases (*P *= 0.026) (Table 3). However, a significant difference was not evident in either the Oxford or WTCCC case sample set (*P *= 0.55 and 0.80, respectively) (Table 3).

**Table 3:** Subphenotype analysis of *rs3738919 *in rheumatoid arthritis patients

	CC, number (frequency)	CA, number (frequency)	AA, number (frequency)	*P *value
New Zealand				
Gender				
Male	117 (0.500)	90 (0.385)	27 (0.115)	0.026
Female	177 (0.394)	216 (0.481)	56 (0.125)	
RF				
Yes	232 (0.431)	239 (0.444)	67 (0.125)	0.75
No	49 (0.441)	51 (0.459)	11 (0.100)	
SE				
Yes	241 (0.418)	269 (0.467)	66 (0.115)	0.05
No	75 (0.503)	53 (0.356)	21 (0.141)	
Oxford, UK				
Gender				
Male	89 (0.434)	86 (0.420)	30 (0.146)	0.55
Female	211 (0.416)	233 (0.460)	63 (0.124)	
RF				
Yes	227 (0.411)	247 (0.447)	78 (0.141)	0.34
No	69 (0.454)	68 (0.447)	15 (0.099)	
SE				
Yes	226 (0.410)	254 (0.461)	71 (0.129)	0.47
No	74 (0.457)	66 (0.407)	22 (0.136)	
WTCCC				
Gender				
Male	180 (0.438)	187 (0.455)	44 (0.107)	0.80
Female	528 (0.423)	574 (0.460)	146 (0.117)	

RF, rheumatoid factor; SE, shared epitope; WTCCC, Wellcome Trust Case Control Consortium.

## Discussion

There was no evidence supporting a role for the *ITGAV *SNP *rs3738919 *in the etiology of RA when the NZ and the Oxford case control sample sets were analysed separately (*P *= 0.11 and 0.07, respectively) (Table 1). Trends for association were observed in both sets of samples, but in opposing directions (OR = 0.86 and 1.18, respectively). When all of the available sample sets were analysed together in a random effects model owing to the heterogeneity caused by the Oxford sample set, there was no longer evidence for an association of *rs3738919 *with RA (OR = 0.92; *P *= 0.29). To further investigate a potential association of RA with *rs3738919*, genotyping in a very large cohort will be required. The work previously undertaken by Thomson and colleagues [8] in confirming an association with RA of the Chr6q23 locus is an excellent example of how this can be achieved. A sample set of this size would have 69% power (α = 0.05; OR = 0.92) to detect association at *rs3738919*.

The rs3738919 *ITGAV *variant previously showed no association with RA in Japanese case-control samples [28]. These data were not included in our meta-analysis given that the major allele was present at a considerably different frequency in Japanese controls (0.92) than in Caucasian controls (France, 0.58; OXFORD, 0.64; and NZ, 0.63). It is already clear that there are genetic differences in susceptibility to RA between the Japanese and Caucasian populations. For example, the HLA-DRB1*0405 allele is most strongly associated with RA in Japanese patients [29] whereas the *0401 and *0404 alleles are more associated with RA in Caucasians [30]. Other genes showing population-specific effects in RA are the R620W variant of the protein tyrosine phosphatase, *PTPN22*, associated with Caucasian RA [4,5] but monomorphic in the Japanese population [31]; *CTLA4*, associated with RA in Caucasian [6] but not associated with RA in Japanese patients [32]; peptidylarginine deiminase type 4, *PADI4*, associated in Japanese patients [33,34] but very weakly in Caucasians [6,35,36]; and the Fc receptor-like 3 gene variant, *FCRL3-169C*, associated with RA in Japanese patients [37,38] and in one Caucasian study [39] but not in other Caucasian studies [40,41]. In conclusion, we have not been able to provide further support for the involvement of *ITGAV *in the etiology of RA in Caucasians. However, it is important that further genotyping be done in a large independent cohort to confirm whether *ITGAV *plays a role in RA.

## Conclusions

In Caucasians, meta-analysis of 3,527 cases and 4,126 controls does not provide further evidence for a role of the *ITGAV *SNP *rs3738919 *in the development of RA.

## Abbreviations

ACR: American College of Rheumatology; CI: confidence interval; CTLA4: cytotoxic T-lymphocyte antigen 4; ITGAV: integrin ανβ3; NZ: New Zealand; OR: odds ratio; OR_*allelic*_: odds ratio for allele frequency difference; PTPN22: protein tyrosine phosphatase non-receptor 22; RA: rheumatoid arthritis; RF: rheumatoid factor; SNP: single-nucleotide polymorphism; WTCCC: Wellcome Trust Case Control Consortium.

## Competing interests

The authors declare that they have no competing interests.

## Authors' contributions

JEH-M and TRM helped to design the study, oversee its execution, and prepare the manuscript. KAR, AJP-G and MEM provided technical support. PG, AAH, PBBJ, LKS and PH helped to provide clinical recruitment and analyse data. ND, JH and BPW helped to provide clinical recruitment, analyse data, and prepare the manuscript. All authors read and approved the final manuscript.

## Supplementary Material

Additional file 1 Allele and genotype distribution of *rs10174098 *and *rs3911238*. OR, odds ratio; CI, confidence interval; HWE, Hardy-Weinberg equilibrium; UK, United Kingdom; WTCCC, Wellcome Trust Case Control Consortium. Click here for file
