# Supplementary material for: The ITGAV rs3738919 variant and susceptibility to rheumatoid arthritis in four Caucasian sample sets
Source: Arthritis Res Ther. 2009 Oct 9;11(5):R152. doi: 10.1186/ar2828 (PMC2787269; doi:10.1186/ar2828)
Supplement: Additional file 1 — Allele and genotype distribution of rs10174098 and rs3911238. OR, odds ratio; CI, confidence interval; HWE, Hardy-Weinberg equilibrium; UK, United Kingdom; WTCCC, Wellcome Trust Case Control Consortium. [file ar2828-S1.DOC]

**Supplementary Table 1** Allele and genotype distribution of *rs10174098* and *rs3911238.* OR, odds ratio; CI, confidence interval; HWE, Hardy-Weinberg equilibrium; UK, United Kingdom; WTCCC, Wellcome Trust Case Control Consortium.

|  | ***rs10174098*** | | | | ***rs3911238*** | | | |
| --- | --- | --- | --- | --- | --- | --- | --- | --- |
| **Cohort** | **Case (freq)** | **Control (freq)** | ***P*** | **OR [95% CI]** | **Case (freq)** | **Control (freq)** | ***P*** | **OR [95% CI]** |
| **New Zealand** |  |  |  |  |  |  |  |  |
| **Minor allele** | 478 (0.324) | 336 (0.325) | 0.97 | 1 [0.85-1.19] | 392 (0.266) | 253 (0.248) | 0.36 | 1.10 [0.92-1.32] |
| **Genotype 1,1** | 341 (0.46) | 234 (0.45) | - | 1 | 398 (0.54) | 288 (0.57) | - | 1 |
| **1,2** | 316 (0.43) | 230 (0.45) | 0.63 | 0.94 [0.74-1.20] | 286 (0.39) | 191 (0.38) | 0.51 | 1.08 [0.85-1.37] |
| **2,2** | 81 (0.11) | 53 (0.10) | 0.81 | 1.05 [0.71-1.54] | 53 (0.07) | 31 (0.06) | 0.37 | 1.24 [0.77-1.98] |
| **HWE** | 0.55 | 0.75 |  |  | 0.87 | 0.93 |  |  |
| **Dominant** |  |  | 0.74 |  |  |  | 0.39 |  |
| **Additive** |  |  | 0.95 |  |  |  | 0.32 |  |
| **Recessive** |  |  | 0.68 |  |  |  | 0.44 |  |
| **Oxford, UK** |  |  |  |  |  |  |  |  |
| **Minor allele** | 441 (0.315) | 333 (0.323) | 0.65 | 0.96 [0.81-1.14] | 344 (0.270) | 272 (0.267) | 0.88 | 1.01 [0.84-1.22] |
| **Genotype 1,1** | 322 (0.46) | 240 (0.47) | - | 1 | 335 (0.53) | 275 (0.54) | - | 1 |
| **1,2** | 316 (0.45) | 217 (0.42) | 0.50 | 1.09 [0.85-1.38] | 256 (0.40) | 196 (0.39) | 0.58 | 1.07 [0.84-1.37] |
| **2,2** | 62 (0.09) | 58 (0.11) | 0.26 | 0.80 [0.54-1.18] | 43 (0.07) | 38 (0.08) | 0.76 | 0.93 [0.58-1.48] |
| **HWE** | 0.20 | 0.40 |  |  | 0.49 | 0.71 |  |  |
| **Dominant** |  |  | 0.84 |  |  |  | 0.69 |  |
| **Additive** |  |  | 0.64 |  |  |  | 0.89 |  |
| **Recessive** |  |  | 0.17 |  |  |  | 0.65 |  |
| **WTCCC** |  |  |  |  |  |  |  |  |
| **Minor allele** | 1167 (0.314) | 1928 (0.328) | 0.14 | 1.07 [0.98-1.17] | 994 (0.267) | 1478 (0.252) | 0.09 | 1.09 [0.99-1.19] |
| **Genotype 1,1** | 891 (0.48) | 1324 (0.45) | - | 1 | 1018 (0.55) | 1664 (0.57) | - | 1 |
| **1,2** | 771 (0.42) | 1300 (0.44) | 0.044 | 1.13 [1.00-1.28] | 690 (0.37) | 1070 (0.36) | 0.40 | 1.05 [0.93-1.19] |
| **2,2** | 198 (0.11) | 314 (0.11) | 0.52 | 1.07 [0.88-1.30] | 152 (0.08) | 204 (0.07) | 0.085 | 1.22 [0.97-1.52] |
| **HWE** | 0.11 | 0.85 |  |  | 0.023 | 0.076 |  |  |
| **Dominant** |  |  | 0.055 |  |  |  | 0.20 |  |
| **Additive** |  |  | 0.14 |  |  |  | 0.093 |  |
| **Recessive** |  |  | 0.96 |  |  |  | 0.11 |  |
| **Jacq [21]** |  |  |  |  |  |  |  |  |
| **Minor allele** | NA | NA | NA | NA | NA | NA | NA | NA |
| **Genotype 1,1** | NA | NA | NA | NA | NA | NA | NA | NA |
| **1,2** | NA | NA | NA | NA | NA | NA | NA | NA |
| **2,2** | NA | NA | NA | NA | NA | NA | NA | NA |
| **HWE** | NA | NA |  |  | NA | NA |  |  |
| **Dominant** |  |  |  |  |  |  |  |  |
| **Additive** |  |  |  |  |  |  |  |  |
| **Recessive** |  |  |  |  |  |  |  |  |
| **Combined** |  |  |  |  |  |  |  |  |
| **Minor allele** | 2086 (0.316) | 2597 (0.327) | 0.16 | 0.95 [0.88-1.02] | 1730 (0.267) | 2003 (0.253) | 0.051 | 1.08 [1.00-1.16] |
| **Genotype 1,1** | 1554 (0.47) | 1798 (0.45) | - | 1 | 1752 (0.54) | 2229 (0.56) | - | 1 |
| **1,2** | 1405 (0.43) | 1750 (0.44) | 0.14 | 0.93 [0.84-1.02] | 1235 (0.38) | 1458 (0.37) | 0.15 | 1.08 [0.98-1.19] |
| **2,2** | 341 (0.10) | 426 (0.11) | 0.36 | 0.93 [0.79-1.09] | 248 (0.08) | 273 (0.07) | 0.13 | 1.15 [0.96-1.38] |
| **HWE** | 0.36 | 0.98 |  |  | 0.14 | 0.097 |  |  |
| **Dominant** |  |  | 0.12 |  |  |  | 0.071 |  |
| **Additive** |  |  | 0.15 |  |  |  | 0.051 |  |
| **Recessive** |  |  | 0.59 |  |  |  | 0.21 |  |
